# The role of neuro-supportive substances of natural origin in neurological conditions—A literature-based formulators' perspective

**DOI:** 10.3389/fneur.2025.1647092

**Published:** 2025-12-09

**Authors:** Roy van Brummelen, Anna C. van Brummelen

**Affiliations:** Van Brummelen Consultants, Pretoria, South Africa

**Keywords:** stroke, Alzheimer's, Parkinson's, antioxidants, inflammation, resveratrol, curcumin, citicoline

## Abstract

Products of natural origin are seldom tested up to a point of full acceptance, mainly due to a lack of financial viability for commercialization. Yet many come with a rich history of use and proof of concept testing. We investigated literature regarding the possible role and function of the best known of these nutraceuticals in relationship to three neurological conditions i.e. stroke, Alzheimer's - (AD) and Parkinson's disease (PD), and their potential as supportive therapies. Current studies suggest that citicoline has a neuroprotective effect in ischemic conditions, playing a role in the restoration of the barrier function of endothelial cells, activating repair mechanisms and possibly decreasing ischemic lesion size in stroke, as well as increasing dopamine availability in PD. Citicoline was also demonstrated to increase the levels of sirtuin 1 (SIRT1), thus reducing inflammation—leading to improved cognitive status and a better quality of life in cognitive impairment. N-Acetylcysteine (NAC) shows pro-cognitive effects, increasing glutathione (GSH) levels that are decreased in AD and PD patients, possibly decreasing neuroinflammation. Mechanistic studies indicate the potential neuroprotective and neurorestorative effects of resveratrol by its anti-inflammatory and anti-apoptotic activity, also increasing SIRT1 levels and promoting the outgrowth of neurite protrusions and synaptogenesis. Curcumin's anti-inflammatory effects via inhibition of interleukin 1 (IL-1) and tumor necrosis factor-alpha (TNF-alpha) can potentially delay progression of PD. Some nutraceuticals, e.g., citicoline, show synergism in combination with current therapies. We propose a renewed, risk-benefit approach for inclusion of the investigated nutraceuticals with limited indications in certain neurological treatment regimens.

## 1 Introduction

### 1.1 Acceptance of treatments of natural origin

The challenge of “lack of novelty” makes most natural substances and formulas not patentable. This means that full clinical trials to prove safety and efficacy, as prerequisite for regulatory registration and acceptance by the medical community, seldom take place (1). Simply put, very few companies are prepared or able to invest the estimated $515.8 million cost (2018 data) (2) required to do these types of studies with no possibility of patent protection to recover their costs. However, the lack of testing does not necessarily reflect a lack of efficacy, but often rather a lack of financial viability for commercialization.

The purpose of this perspective is to investigate the possible augmenting role of five specific natural substances in cognitive neurological conditions namely stroke, Alzheimer's - (AD) and Parkinson's disease (PD), based on existing and developing research found in the literature. The goal is to propose a novel approach for the controlled inclusion of some of these substances as adjuvant therapies in current treatment regimens on a risk-benefit ratio principle.

## 2 Therapeutic targets of stroke, Alzheimer's - and Parkinson's disease

### 2.1 Stroke

Stroke is a complex disorder that involves the activation of several harmful signaling cascades in the ischemic core and surrounding penumbra of the stroke event. A multi-targeted approach with various agents that can intervene on different levels is required to have a neuroprotective effect that is effective (3). The neuroinflammation following an ischemic episode plays a major role in the subsequent damage and cell death and needs to be prevented. The chain reaction below, summarized by Maida et al. (4), follows the initial ischemic neuronal damage:

- The release of glutamate, which leads to excessive activation of N-methyl-D-aspartate (NMDA) receptors and a heavy flow of Ca2+ into cells, leading to their death due to excitotoxicity.
- Damaged neurons and astrocytes produce reactive oxygen species (ROS) and depletes glutathione (GSH), an essential antioxidant.
- This oxidative stress sets off the inflammatory process which contributes to the rupture of the blood-brain barrier (BBB).
- This allows activated blood-borne immune cells to reach the cerebral parenchyma and accumulate in the ischemic tissue with the subsequent activation of microglia, the macrophages of the central nervous system (5).
- The activated microglia secrete proinflammatory agents e.g., cytokines such as tumor necrosis factor-alpha (TNF-alpha), ROS and nitric oxide, which is detrimental, although microglia can also have positive effects i.e., removing dead tissue and debris.
- The damaged brain tissue leads to further activation of microglia and infiltrating leukocytes and the release of more inflammatory cytokines.
- This promotes the expression of adhesion molecules on endothelial cells and the recruitment of more leukocytes from the peripheral blood (4).

These successive reactions that follow the ischemic incident lead to increased neuronal death and a larger area of infarction that worsen the neurological outcome (4).

### 2.2 Alzheimer's disease

AD is a slow, progressive, neurodegenerative disease, characterized by the accumulation of neuritic or amyloid plaques outside neurons and neurofibrillary tangles inside neurons. These amyloid plaques are extracellular deposits of beta-amyloid protein, and the neurofibrillary tangles are abnormal, hyperphosphorylated tau protein filaments (6). There are two main hypotheses behind the neurodegeneration that causes AD; the cholinergic hypothesis and the amyloid hypothesis (6).

The brain uses 20% more oxygen than any other organ and is therefore more exposed to ROS and reactive nitrogen species (RNS). ROS and RNS are both very unstable and interact with neurons, since they contain large amounts of polyunsaturated fatty acids—this results in lipid oxidation, altered redox potential of beta-amyloid metal ions as well as mitochondrial dysfunction, leading to apoptosis of the neuron. The oxidative stress and breaking of bonds within DNA molecules accelerate aging and neuronal death. Many products of these reactions lead to more inflammatory mediators [e.g., interleukin 1 (IL-1) and TNF-alpha] causing further neuroinflammation in the brain of the Alzheimer's patient (7).

Neuroinflammation is a major part of the pathology of AD (8). One of the target areas of new drug development, is to reduce beta-amyloid toxicity by inhibiting the excessive activation of microglia (including their excessive phagocytosis of the amyloid plaques)—which is also a source of even more pro-inflammatory cytokines causing inflammation of healthy neurons in the AD patient (7). In AD neuroinflammation, cyclooxygenase-2 (COX-2) levels are often elevated and reflect beta-amyloid levels—elevated COX-2 also causes the formation of free radicals and further neuroinflammation (7).

### 2.3 Parkinson's disease

Parkinson's is a neurodegenerative disorder characterized mainly by a lack of dopamine due to dopaminergic neuronal cell death (9). The ultimate underlying reason for the dopaminergic death is still uncertain. Some of the risk factors are also closely linked with inflammation, which is largely suspected to be involved and important in the development of PD. It is likely that abnormally folded or phosphorylated alpha-synuclein (p-syn) brain protein plays a direct role (10) since its aggregation is a common finding in PD. These aggregates can damage dopaminergic neurons and cause the formation of Lewy bodies (LB) and eventual necrosis. The LB can trigger a chain reaction of events. In a non-pathological state, LB aggregates are usually scavenged by a proteasome complex or lysosome. However, defects in these scavenging pathways are common in PD, which causes a further spread of aggregates (11).

Therapeutic targets in PD have mainly been maintaining dopamine levels by either the inhibition of endogenous dopamine degradation (e.g., via monoamine oxidase B inhibitors) or by supplying levodopa or dopamine agonists. Recently, inhibition of the inflammation processes such as inhibiting microglial activation or IL-1 and TNF-alpha, found to be elevated in the striatum of PD patients, have been receiving focus (10).

## 3 Natural treatments for stroke, Alzheimer's - and Parkinson's disease

### 3.1 Citicoline/cytidine-5′-diphosphocholine

Citicoline or cytidine-5′-diphosphocholine (CDP-choline) is an endogenous compound that provides choline for the biosynthesis of acetylcholine (ACh), and it is the natural intracellular precursor of the phospholipid phosphatidylcholine—essential in the synthesis of cell membranes (including neuronal membranes). The cytidine fraction is transformed into uridine and then used for DNA and RNA synthesis, as well as for the synthesis of membrane components and glycosylation (12).

In a prospective clinical study with stroke patients, citicoline was demonstrated to increase norepinephrine and dopamine levels in the central nervous system and to have a potential neuroprotective effect in ischemic conditions (13). Also, citicoline seems to restore the activity of mitochondrial ATPase and membrane Na+/K+ATPase, it inhibits activation of phospholipase A2 (and the production of inflammatory mediators) and it accelerates the reabsorption of cerebral oedema in several experimental models (14).

The proposed neuroprotective effects of citicoline can feasibly be explained by the restoration of the barrier function of endothelial cells, specifically the BBB, and the inhibition of mitochondrial permeability (which, if increased, can lead to cell death) as well as better neuronal membrane integrity. Citicoline also increases the levels of sirtuin 1 (SIRT1), a NAD+ dependent deacetylase, reducing inflammation (12) and having an anti-platelet aggregation effect (15). Citicoline was shown to act synergistically with thrombolytic and neuroprotective drugs in an animal model (12, 16).

However, a large human study (ICTUS trial with *n* = 2,298) found no significant recovery rates between the active and placebo groups of patients with moderate to severe stroke. Subgroup analyses hinted at possible benefit in less severe stroke [patients older than 70 years, not treated with a plasminogen activator within 4½ h of symptom onset], but the results were not conclusive (17). According to a review by Overgaard, citicoline has been extensively studied in clinical trials with more than 11,000 patients and appears to be most beneficial in less severe stroke patients. In these patients citicoline may have some neuroprotective effects (15).

New publications report citicoline as a safe, neuroprotective agent especially in mild cognitive impairment, that is well-tolerated (12, 15, 18, 19), with clinical trial evidence in acute ischemic stroke (especially less severe stroke) (13, 20) and other neurological disorders; as well as presenting domain-specific benefits particularly in cognitive recovery and quality of life (14, 21). Long term citicoline treatment after a first ischemic stroke is associated with an improved cognitive status and a better quality of life up to 2 years after the incident (22). Further studies in stroke need to focus more on specific subgroups such as mild stroke and pediatric subgroups, which shows promise (23), also combination therapies and the optimizing of its role as an augmentative treatment.

In a retrospective case-control AD study by Gareri et al. (24) with 448 patients, citicoline combined with an ACh-esterase inhibitor (AChEI i.e., donepezil, rivastigmine or galantamine) was shown to be more effective than the AChEI alone to slow down cognitive impairment progression. A similar study in older patients demonstrated that triple therapy with citicoline, memantine, and an AChEI was more effective in maintaining the total cognitive examination score for 12 months, than memantine and the AChEI alone (25). Other neuro-immunomodulatory and neurophysiological benefits in AD patients have also been reported (26), such as the normalization of IL-1β and histamine levels, lessening inflammation. Citicoline was also shown to increase cerebral blood flow (12). Follow up trials to confirm these augmenting effects of citicoline in AD should be performed, as well as to elucidate the mechanism of action and optimize the dosage requirements.

In PD, citicoline has shown capacity to increase dopamine availability in the striatum and to act as a dopaminergic agonist in various experimental Parkinson's models, thereby improving bradykinesia, rigidity and tremors (27). The increase in dopamine levels is partly due to an increase in dopamine synthesis (through tyrosine hydroxylase activation) and the inhibition of dopamine reuptake (27). Chronic citicoline administration, according to more than one review, seems to promote partial recovery of dopaminergic and muscarinic receptors that decrease with aging (12, 28). Citicoline appears to be an effective treatment for PD in both untreated patients and patients already treated with levodopa, in whom it allows for reducing the levodopa dosage (12), showing promise as an adjuvant therapy in PD (27).

The oral bioavailability of citicoline is very good (often > 90%) and it is well tolerated with dosages of 500–2,000 mg/day (12, 21).

### 3.2 Glutathione & N-acetylcysteine

Many antioxidants exert their action by increasing the levels of GSH—a potent, naturally occurring intracellular antioxidant. Oral GSH supplementation, however, is not effective since it is quickly hydrolyzed in the liver and intestines and has a limited capacity to cross the BBB. N-acetylcysteine (NAC) supplies L-cysteine, the rate limiting step in the formation of GSH (29), and animal models have shown that NAC effectively penetrates the BBB, increasing GSH levels in the brain, as described in a review by Tardiolo et al. (30). A study in PD patients demonstrated that NAC crosses the BBB in humans (31), including children, when combined with probenecid (32). NAC treatment was shown to cause a 50% decrease in infarct volume area and an increase in the neurology score in an animal stroke model (30).

AD patients have decreased GSH levels and formulations containing NAC have shown pro-cognitive benefits for these patients (33, 34). NAC also protects against beta-amyloid toxic effects and promotes the inhibition of nuclear factor-kappa B and inducible nitric oxide synthase enzyme, decreasing inflammatory cytokines and neuroinflammation (30).

Regarding PD, intravenous administration of GSH showed significant improvements in nine patients which lasted 2–4 months after concluding the therapy (35). NAC supplementation (in animal models) leads to an increase of GSH brain levels, a reduction in oxidative damage, increased brain synaptic and non-synaptic connection, as well as mitochondrial complex I expression. All of which should lead to reduced cell death (30). Optimum dosages for specific conditions and improved dosage forms need to be further investigated. NAC seems to have a low risk for pro-oxidant effects, but no reported cases could be found in the literature at clinically used dosages. Dosages of 600–3,000 mg/day are used chronically with a well-established safety profile and toxicity uncommon (36, 37).

### 3.3 Resveratrol

Resveratrol (3,5,4'-trihydroxytrans-stilbene) is a natural polyphenol (38). Resveratrol showed potential in animal models to be neuroprotective, as well as neuro-restorative—via anti-inflammatory and anti-apoptotic effects and by promoting the outgrowth of neurite protrusions and synaptogenesis (39). Resveratrol supplementation reduced ischemic cell death in an animal model with a second or recurrent, mild stroke (40). Lopez et al. (41) also concluded that resveratrol prevents secondary damage after stroke and that pretreatment with resveratrol is an option to induce ischemic tolerance. A meta-analysis in rodents described that resveratrol reduces BBB disruption and oedema, thus supporting resveratrol treatment as a possible low risk strategy to protect the brain from enhanced damage after an ischemic event (40, 42).

There are various mechanisms postulated by which resveratrol assists the brain during ischemia:

- By preventing thrombotic events via suppressing cyclooxygenase-1 (COX-1) derived thromboxane A2 production in platelets and by inhibiting platelet Ca2+ influx (43).
- By regulating platelet activation by influencing vasodilator-stimulated phosphoprotein signaling (44) involving actin dynamics, integrin activation and platelet aggregation (45).
- By affecting SIRT1 by activating it to exert its neuroprotective effects (46).
- By inhibiting monomeric C-reactive protein induced activation of COX-2, thereby inhibiting the release of proinflammatory cytokines (47).
- Resveratrol seems to mimic the effect of ischemic preconditioning, a powerful endogenous mechanism of protection (48)—it could inhibit activation of microglia and inflammation and so exert a neuroprotective effect after stroke (39).
- It helps maintain the integrity of the BBB via reduction of matrix metalloproteinase-9, involved in the degradation of the extracellular matrix, and induces adaptive immune responses and delay neuronal death (49).
- Resveratrol is furthermore postulated to enhance the clearance of beta-amyloid peptides and so limit neuronal damage (50).

A review of PD rodent models corroborates that resveratrol has obvious neuroprotective effects (51) and it prevents behavioral as well as neurological disorders in *in vitro* and *in vivo* experimental PD models (52).

Although evidence for clinically significant pro-oxidant effects of resveratrol is limited in humans, the possible risk of pro-oxidation at high dosages of more than 1 g/day must be guarded against (53, 54), also the possible inhibition of cytochrome P450 enzymes and the effect on the metabolism of anticonvulsants, antidepressants and other neuroactive drugs (54). Dosages of 250–500 mg/day are seen as typical safe ranges (55, 56), with side effects only seen at dosages >2,5 g/day (53). Resveratrol is also notorious for being over-promising in *in vitro* and *in vivo* animal models but underperforming in human studies due to bioavailability issues—not due to absorption (up to 75%), but mainly due to rapid metabolism in combination with some solubility and stability issues. New research focuses on nano-encapsulation and casein nanoparticles (liposomes, solid lipid nanoparticles, phospholipid complexes etc.) that achieve up to 100-fold higher bioavailability; cyclodextrin, protein and polymer complexes; as well as chemical derivatisation (methoxylated, hydroxylated and halogenated derivatives) (57, 58). Of special interest is the increased efficacy achieved in combination with bio-enhancers/metabolism inhibitors (e.g., piperine) and co-administration with other natural substances such as other polyphenols (e.g., curcumin) (59). The combination of different polyphenols seems to have synergistic therapeutic effects, possibly due to enhanced bioavailability of polyphenol complexes with better stabilized structures and availability for absorption, in addition to cooperative effects (59). If bioavailability can be sufficiently addressed, resveratrol holds great potential as neuroprotective agent in humans.

### 3.4 Curcumin

Curcumin (diferuloylmethane) is also a polyphenol and the active ingredient of the spice turmeric, derived from *Curcuma longa* root (60). A systematic review of curcumin in 2022 concluded that its neuroprotective, antioxidant and anti-inflammatory effects indicate that it could be a possible therapeutic option for stroke patients (61). This was subsequently confirmed by a molecular dynamics study (62).

An extensive review of curcumin analogs, derivatives and hybrids' therapeutic, preventative and diagnostic applications in AD, designated curcumin as a substance with antioxidant, anti-inflammatory and anti-amyloid activity, as well as an inhibitor of AChE (63). Its bioavailability issues and lack of selectivity, however, will cause newer studies to focus on curcumin derivatives (63).

*In vitro* and *in vivo* results from PD models have shown that curcumin targets multiple degenerative pathways such as oxidative/nitrosative stress, mitochondrial dysfunction, protein aggregation and restoring neuronal function in the substantia nigra—thereby helping to restore striatal dopamine levels (64). Curcumin have strong inhibitory effects against IL-1 and TNF-alpha, thus has potential to treat the inflammation involved in the onset and progression of PD (65). Curcumin was also shown to improve levels of the reduced form of GSH and to prevent abnormally folded p-syn aggregates in a lipopolysaccharide-induced rat model of PD (66). Donadio et al. (67) corroborated curcumin's decrease of misfolded, pathological p-syn in skin nerves of PD patients as supported by multiple regression models. They also showed that curcumin's active metabolite can cross the BBB, when formulated with phospholipids (67). PD patients receiving chronic curcumin supplementation furthermore showed an improved clinical evaluation according to the non-motor symptoms scale, while those of the controls were worse, even though their L-dopa dosage was increased (67). A review publication by Jin et al. (68) described that curcumin could possibly delay the progression of PD by activating the brain-derived neurotrophic factor and the phosphatidylinositol-3 kinase/protein kinase B signaling pathways, involved in nerve regeneration and anti-apoptosis, to mediate neuroprotection.

Due to its antioxidant actions, the risk of pro-oxidant effects at high dosages of more than 1–2 g/day must be avoided, as this could lead to oxidative stress, DNA damage and neurocognitive deficits, according to some reports (54). Similar to resveratrol, the possible inhibition of cytochrome P450 enzymes by curcumin and the effect on the metabolism of anticonvulsants, antidepressants etc., must be kept in mind when treating patients with neurological disorders (54). Dosages of 500 mg/day are seen as typical safe ranges (56), with dosages up to 1,600 mg/day used in studies and even as high as 8 g/day for short periods (69). Like resveratrol, curcumin also has bioavailability issues and new research focuses on nano-formulations; cyclodextrin, protein and polymer complexes; as well as chemical derivatisation. Increased efficacy is also being achieved by combination with other polyphenols and bio-enhancers/metabolism inhibitors (e.g., piperine), increasing bioavailability up to 2,000% (70, 71).

### 3.5 Green tea

Green tea extract and its main active, the polyphenol epigallocatechin-3-gallate (EGCG), were shown to reduce spatial and reference memory loss due to ischemic damage (72) and to decrease brain infarct size (in an animal model) (73). It also increases GSH, explaining its possible neuroprotective effects through its antioxidant properties (48). EGCG also decreases the proteases, beta- and gamma-secretase, that play a crucial role in the production of beta-amyloid peptides. It thereby reduces the beta-amyloid levels and could thus prevent neuronal death (74). In summary of the studies reviewed by Uddin et al., they concluded that EGCG is useful in the treatment of neurodegenerative diseases and can prevent cognitive dysfunction. EGCG can furthermore assist with restoring mitochondrial functions and can help to prevent synaptic functional loss (74).

Green tea is considered safe but high dosages of EGCG of more than 600 mg/day have been associated with potential pro-oxidant activity and hepatotoxicity. Green tea extract dosages of 250–1,000 mg (containing 100–400 mg EGCG) are still considered safe (75).

## 4 Discussion

### 4.1 Strengths

The natural substances discussed in this publication have been known and used for many years. A rich history of use does not proof safety, but it does give an important preliminary indication if no major problems presented itself over years of extensive use in a wide part of the world's population. Safety data for most of the nutraceuticals discussed here, as single ingredients, are already available to a significant extent (12, 21, 36, 37, 55, 56, 69, 75).

New studies regularly propose these specific nutraceuticals as possible supportive or adjuvant therapies in the treatment of minor stroke, AD and PD; specifically, for limiting and preventing neurological damage—although most not quite at phase IV clinical trial level yet (21, 24, 33–35, 51, 52, 63, 65–67, 74).

The substances are readily available, as they are often also used for other indications and at reasonable cost, which make them attractive as possible add-ons to current treatment regimens. Preliminary work, indicating that some act synergistically and/or in support of current treatments, are also available (24, 25).

### 4.2 Challenges

Due to species differences, results of animal models alone can sometimes fail to translate to clinical benefit—this holds true especially for certain preliminary work done with polyphenols i.e., resveratrol in cancer. This also relates to differences in pharmacokinetics and pharmacodynamics issues e.g., absorption, the influence of gut microbiota and metabolism. Much work still needs to be done on bioavailability, especially on the polyphenols, but progress is being made (57, 58, 70, 71).

The biggest barriers to clinical translation, scientifically and regulatory, are probably the standardization of dosages (or the lack there-of), the use of multiple combination products, and varying levels of quality control between the various manufacturers. This, together with the lack of financial feasibility to do the necessary studies, as explained in paragraph 1.1, remain the most problematic.

Regarding effective dosages, much more work needs to be done, especially in respect to combination products. For single ingredient products, some data regarding dosages are available and generally fall well within considered safe or well tolerated levels. Higher dosages often do not prove more effective with contrasting results. For example, early work by Clark et al. (76) on citicoline showed results with 500 mg/day being more effective than 2,000 mg/day in their trials. This was, however, in contrast with a later study (2009) which found citicoline at 2,000 mg/day to be more effective (77). Similarly, resveratrol has been used as high as 2,000 mg/day (78), but long-term cognitive benefits were observed at much lower dosages (75–200 mg/day) (79). With respect to NAC, clinical trials of PD and cognitive impairment have successfully used 1,200–2,400 mg/day, with some evidence of neuroprotective effects (30), still within its safety profile, as discussed in paragraph 3.2. With curcumin, large variations of dosages have been used, depending on the bioavailability of the specific form of curcumin used—with dosages of 500 mg up to 1,600 mg and even higher per day (56, 69). EGCG still require more and larger studies to determine effective dosages (67). Generally, most of these substances seem to have a wide therapeutic index and it would be wise to err on the conservative side to avoid any possibility of pro-oxidant effects.

### 4.3 Potential

The nutraceuticals summarized in this publication offer a significant chance of benefit with relatively low risk in support of conditions with limited alternatives. The still largely untapped biodiversity of plants worldwide as a source of more substances such as these and the rich history of use from different population groups all over the world, i.e., African, Chinese, Indian, South American etc., hold so much potential that still needs to be unlocked.

### 4.4 Recommendations

More studies, especially by NGO's, government and research institutions, focusing on some of the gaps highlighted above, should be encouraged. These organizations could focus specifically on single ingredient products. We recommend that for beneficial nutraceutical substances, such as these discussed, limited claims within safe dosage ranges should be identified and allowed in certain conditions by the regulatory authorities. This could for example be done on the risk-benefit principle proposed, based on strong phase II clinical data (still with the necessary quality and safety data) combined with phase IV (post-marketing) ongoing safety/efficacy monitoring. Phase III studies, the biggest limitation regarding cost, would then in such cases, not be a prerequisite. These claims could be allowed only to health care professionals and not directly to the public. If a company intends to register a combination product containing more than one of these substances, they could be required to apply under the same conditions, but with additional phase II studies showing superior efficacy or benefit above the single ingredient. If the claim is accepted, it will be approved only for the specific/trademarked product and dosage which complied to these additional testing requirements.

This limited form of protection for these companies will help to make these studies more financially viable and thus encourage more of the necessary research in this field. Special attention to continued education will also be required to keep the healthcare profession up to date with the latest research in this field.

### 4.5 Summary

Current research, including proof-of-concept (*in vitro* and *in vivo*), mechanistic and preliminary clinical studies, indicate possible neuro-supportive benefits for the substances discussed at acceptable/safe dosage levels (49, 80, 81), suggesting a possible role as supportive therapies in current treatment regimes.

The possibility that certain polyphenols could work synergistically in both action and bioavailability (59, 82), should be further investigated as a priority. Also, the safety and bioavailability of combinations should be established by further trials, focusing on subgroups of patients with the highest need and best chance of benefit where there are often very few other alternatives.

In our perspective, a new focus on the principle of the risk-benefit ratio is required. Where there are possible advantages for patients affected by neurodegenerative/neuro-damaging conditions at low or no risk and at an affordable price, these natural substances (alone or in combination) should be included in treatment regimens under the supervision of healthcare professionals. This can be made possible by a form of expedited or limited registration as discussed, still with important and necessary attention to quality and safety. Trademarks rather than patent protection would thus be used, with limited claims allowed to be communicated to healthcare professionals only. This limited form of protection for tested, trademarked products with sufficient data, confirming benefit of their product, could open the door for some form of financial viability to do such studies, even if limited.

If not, many nutraceuticals with potentially huge impact, may never be considered—not due to lack of efficacy, but simply due to a lack of extensive testing (to conclusively confirm full medical indication for pharmaceutical registration), resulting from a lack of financial viability for commercialization.

## Data Availability

The original contributions presented in the study are included in the article/supplementary material, further inquiries can be directed to the corresponding author.
